# Post-COVID-19 fatigue: the contribution of cognitive and neuropsychiatric symptoms

**DOI:** 10.1007/s00415-022-11141-8

**Published:** 2022-04-30

**Authors:** Marco Calabria, Carmen García-Sánchez, Nicholas Grunden, Catalina Pons, Juan Antonio Arroyo, Beatriz Gómez-Anson, Marina del Carmen Estévez García, Roberto Belvís, Noemí Morollón, Javier Vera Igual, Isabel Mur, Virginia Pomar, Pere Domingo

**Affiliations:** 1 Cognitive NeuroLab, Faculty of Health Sciences, Universitat Oberta de Catalunya, Barcelona, Spain; 2 Neuropsychology Unit, Neurology Department, Hospital de la Santa Creu i Sant Pau, Barcelona, Spain; 3 Department of Psychology, Concordia University, Montreal, Canada; 4 Centre for Research On Brain, Language & Music, Montreal, Canada; 5 Facultat de Psicologia, Ciències de l’Educació i l’Esport, Blanquerna, Universitat Ramon Llull, Barcelona, Spain; 6 Internal Medicine Department, Hospital de la Santa Creu i Sant Pau, Barcelona, Spain; 7 Neuroradiology, Department of Radiology, Hospital de la Santa Creu i Sant Pau, Barcelona, Spain; 8 Universitat Autonoma Barcelona (UAB), Barcelona, Spain; 9 Headache Unit, Neurology Department, Hospital de la Santa Creu i Sant Pau, Barcelona, Spain; 10 Psychiatry Department, Hospital de la Santa Creu i Sant Pau, Barcelona, Spain; 11 Infectious Disease Unit, Hospital de la Santa Creu i Sant Pau, Barcelona, Spain

**Keywords:** COVID-19, Fatigue, Cognitive complaints, Neuropsychiatric symptoms, Neuropsychology

## Abstract

Fatigue in its many forms of physical, mental, and psychosocial exhaustion is a common symptom of post-COVID-19 condition, also known as “Long COVID.” Persistent fatigue in COVID-19 patients is frequently accompanied by cognitive dysfunction and neuropsychiatric symptoms; however, less is known about the relationships between these components of post-COVID-19 condition and fatigue itself. Consequently, the present study sought to (1) distinguish the types of fatigue experienced by participants, and (2) investigate whether cognitive deficits across various domains and neuropsychiatric conditions predicted these different types of fatigue. The study included 136 COVID-19 patients referred for neuropsychological evaluation due to cognitive complaints 8 months on average after SARS-CoV-2 infection. Measures included self-reported fatigue (physical, cognitive, and psychosocial), neuropsychiatric questionnaires (assessing symptoms of depression, anxiety, apathy, and executive functioning), a comprehensive neuropsychological assessment, and self-reported quality of life and everyday functioning. Results showed that reports of clinical significant fatigue were pervasive in our sample (82.3% of participants), with physical fatigue rated highest on average relative to the subscale maximum. Elevated levels of apathy, anxiety, and executive dysfunction in neuropsychiatric measures along with executive and attentional difficulties on cognitive tests were found to be consistently important predictors among different types of fatigue. This implicates both cognitive and neuropsychiatric symptoms as predictors of fatigue in post-COVID-19 condition, and stresses the importance of a holistic approach in assessing and considering potential treatment for COVID-19 patients experiencing fatigue.

**Supplementary Information:**

The online version contains supplementary material available at 10.1007/s00415-022-11141-8.

## Introduction

The World Health Organization [1] has recently defined the most common symptoms of post-COVID-19 condition after SARS-CoV-2 infection. Clinical symptoms include fatigue, shortness of breath, and cognitive dysfunction, along with others that impact everyday functioning and are present for at least 2 months during the 3 months after the onset of disease.

Among the first three symptoms mentioned by the WHO, fatigue represents a clinical challenge for at least two reasons. First, there is a lack of a universally accepted definition of fatigue and limited knowledge about its underlying pathogenic mechanism [2]. Defined broadly, fatigue is characterized by excessive tiredness, physical and/or cognitive, and muscular weakness. It has been associated with medical conditions, such as post-viral infection [3] or neurological diseases [4]. However, the distinct manifestations of fatigue depends on the underlying disease as well as sociodemographic variables [5]. Second, it is still unclear whether post-COVID-19 fatigue is associated with cognitive or neuropsychiatric symptoms, as reported in other post-viral conditions (e.g., [6]).

Based on current findings, fatigue has been reported in almost one third of COVID-19 patients, where it is associated with a worse prognosis [7] and can persist at least six months after infection [8]. Reviews on the prevalence of fatigue after COVID-19 estimate that between 9 and 49% of patients show fatigue 4 weeks after symptom onset, while around 30% experience it 12 and 16 weeks after [9, 10]. A recent study even reported that fatigue may persist for 12 months in at least one third of post-COVID-19 patients [11].

After SARS-CoV-2 infection, fatigue can develop as a persistent symptom with impacts on everyday functioning, but it is unclear what clinical factors contribute to it. However, there is convincing evidence that post-COVID-19 fatigue is associated with cognitive and neuropsychiatric symptoms, among other biological and psychological factors [2]. In the present study, we aimed to investigate the nature of post-COVID-19 fatigue by focusing on its relation with these two components. For other clinical conditions, fatigue is associated with decreases in processing speed, sustained attention and executive functions [12]. Indeed, the term ‘cognitive fatigue’ is commonly used to define a condition in which there is impaired performance during tasks requiring a sustained mental effort (also defined as ‘cognitive fatigue’; Chaudhuri and Behan, 2004). Nevertheless, evidence of an association between subjective fatigue and cognitive impairment is mixed, with some studies reporting no association [13–15] and others reporting a link between fatigue and dual-task [16] and executive attention [17].

Looking at the relationship between fatigue and neuropsychiatric disorders, the majority of studies report an association between fatigue and clinical levels of depression and anxiety. These two disorders have been related to fatigue within the context of many neurological conditions ranging from MS [12], post-stroke [18], and neurodegenerative diseases [19]. Despite the evidence that neuropsychiatric symptoms and fatigue are strongly associated, it is important to acknowledge that this association might be partially driven by the similarity of clinical symptoms, especially in people with depression [4]. Additionally, it has been shown that pre-existing diagnosis of depression may increase the severity of fatigue in the months after SARS-CoV-2 infection [11, 20].

At the biological level, neuroimaging studies have found changes in glucose metabolism (in frontal lobe and cerebellum) in COVD-19 patients with fatigue [21], suggesting that neurological sequelae could be contributing to the persistence of this post-infection condition. Additionally, neurophysiological studies have studied the role of the central nervous system in causing fatigue. Ortelli et al. [22] and Versace et al. [23] reported changes in corticomotor inhibition by measuring motor evoked potentials in patients with fatigue after COVID-19. Interestingly, in both studies, patients show clinical symptoms of apathy and cognitive deficits, principally in the domain of executive control, in addition to fatigue.

Taken altogether, these findings suggest a relationship between fatigue, neuropsychiatric and cognitive disorders. However, with the notable exception of Krishnan et al. [24], few studies have investigated whether these three dimensions of the post-COVID-19 condition are interrelated or are independent entities. In the present study, we explored how associated these symptomologies are in a larger sample of patients who were diagnosed with COVID-19 and presented with subjective cognitive complaints on average 8 months after disease onset. First, we assessed the presence of fatigue in its three components (physical, cognitive and psychosocial) with the Modified Fatigue Impact Scale (MFIS, [25]). The impact of fatigue on functional status and quality of life were also assessed with this measure. Second, cognitive status was evaluated with a comprehensive neuropsychological assessment spanning a wide range of cognitive domains, including general cognitive status, attention, short- and long-term memory, language, processing speed, visuo-perceptual–visuo-constructive functions and executive functions. Third, we assessed the presence of neuropsychiatric symptoms including depression, anxiety, apathy and disinhibition. Finally, to investigate the degree to which cognitive and neuropsychiatric disorders predict fatigue, a series of regression analysis were conducted.

Based on previous research, we expected to find a significant relationship between the presence of fatigue (as measured by the total score on the Modified Fatigue Impact Scale) and depression and anxiety [10, 12]. We also hypothesized a significant relationship between fatigue and apathy, given the occurrence of apathy in patients with fatigue after COVID-19 [22]. Considering cognitive functioning, our prediction was less clear, as there is relatively little evidence of a strong relationship between fatigue and cognitive deficits [13–15]. Tentatively, we suspected that the cognitive dimension of fatigue would be predicted by cognitive deficits in attention and executive functioning, as shown by Ortelli et al. [22, 26] and Versace et al. [23]. Additionally, we expected that attention and executive control impairment would contribute to prediction of fatigue, since our previous study found cognitive deficits to be the most prevalent in these domains within a similar sample of COVID-19 patients [27].

## Methods

### Participants

The study included 136 consecutive patients with subjective cognitive complaints after SARS-CoV-2 infection. All patients were evaluated from July 5, 2020 to January 21, 2022 at the Neuropsychology Unit of the Hospital de la Santa Creu i Sant Pau (HSCSP) in Barcelona (Spain). The inclusion criteria for this study were: (a) having had COVID-19 symptoms and confirmed positive for SARS-CoV-2 via polymerase chain reaction (PCR) and/or serology (anti-SARS-CoV2 IgM or IgG); (b) being referred for neuropsychological assessment after reporting subjective cognitive complaints; and (c) being 18 + years old. The exclusion criterion was documented medical history of neurological or psychiatric conditions before the infection.

The sample was composed of 49 males (36%) and 87 females (64%), with a mean age of 51.7 years (SD = 13.5; range 20–88) and mean level of education of 13.6 years (SD = 3.2). The time between the diagnosis of COVID-19 and the neuropsychological assessment was an average of 252 days (SD = 149).

During the course of disease, 59 patients (43.4%) were hospitalized for an average 19.2 days (SD = 17.9) and 24 of them entered the intensive care unit (ICU) (*M* = 18.9 days; SD = 11.2). Non-hospitalized patients were younger (*M* = 47.9; SD = 12.8) than hospitalized patients (*M* = 56.6; SD = 13.1; *p* < 0.001), but with the same number of days elapsed between diagnosis and neuropsychological assessment (Non-hospitalized: *M* = 265.9; SD = 155.4; Hospitalized: *M* = 234.9; SD = 141.1; *p* = 0.24).

### Assessment of fatigue, psychological symptoms, and cognition

All the questionnaires and tests were administered in person, apart from the telephone Montreal Cognitive Assessment (T-MoCA).

*Fatigue* We used the Modified Fatigue Impact Scale (MFIS) to evaluate fatigue in patients [25]. Across 21 items, this questionnaire measures how often fatigue has affected the individual over the past 4 weeks. Each item is scored on a 5-point Likert scale (0: never; 1: rarely; 2: sometimes; 3: often; 4: almost always). The total score ranges from 0 to 84. Moreover, it is possible to calculate three additional subscale scores: physical (score: 0–36), cognitive (score: 0–40), and psychosocial fatigue (score: 0–8). The overall cutoff score is 38 [28].

*Depression and anxiety* We used the Hospital Anxiety and Depression Scale (HADS; [29]) to assess depression and anxiety symptoms. It consists of two sets of seven questions pertaining to each symptomatology. Each item is scored on a 4-point scale ranging from 0 to 3, with the total score ranging from 0 to 42 (or 0 to 21 for each dimension). A score greater than 10 in either depression or anxiety symptoms is considered clinically significant.

*Apathy, disinhibition, and executive functioning* For this study, we used the Frontal Systems Behavior Scale (FrSBe) [30], which measures Apathy (14 items), Disinhibition (15 items) and Executive Dysfunction (17 items). Items are scored in a 5-point scale: 1 (almost never), 2 (seldom), 3 (sometimes), 4 (frequently), 5 (almost always). Responses can be used to quantify behavioral changes over time (before disease and present) on four indices: Apathy, Disinhibition and Executive Dysfunction, and Total Score. *T* scores greater than 65 were considered clinically significant.

*Quality of life and impact on daily functioning* For these areas, we used these three scales:

- European Quality of Life-5 Dimensions (EQ-5D) [31], a self-rating scale includes five items, one for each of the following domains: mobility, self-care, usual activities, pain/discomfort, and anxiety/depression. Items are scored between 1 and 3 (1 = normal, 2 = mildly affected; 3 = severely affected) and the total score can range between 1 and 15. A total score of 5 indicates mild impairment, while 15 indicates severe impairment.
- Brunnsviken Brief Quality of life scale (BBQ) [32], a self-rating scale for subjective quality of life consisting of 12 items. Items are on a 5-step Likert scale, ranging between 0 (full disagreement) and 4 (full agreement). Total score is calculated by multiplying the 2 item scores in each of 6 life areas, and summing these products, for a total score range of 0–96. A total score lower than 52 is considered clinically significant.
- World Health Organization Quality of Life—BREF (WHOQOL-BREF) [33], a self-report questionnaire which assesses quality of life across four domains: physical health (7 items), psychological health (6 items), social relationships (3 items), and environment (8 items). Items are scored between 1 and 5 and all domain scores are normalized to a range of 0–100. A score lower than 60 is considered clinically significant.

*Neuropsychological assessment* Assessment included the following tests: T-MoCA, Conners Continuous Performance Test II (CPT-II), Rey's Auditory Verbal Learning Test (RAVLT), Rey-Osterrieth Complex Figure Test (ROCFT), Digit Span Forward and Backward, Boston Naming Test (BNT), Block Design, Coding, Symbol Search, Trail Making Test A and B (TMT), Stroop task, and verbal fluency tasks (see Supplementary Materials for a complete list of test descriptions and norms used).

### Analysis

We first analyzed the frequency of fatigue, neuropsychiatric and cognitive symptoms to better understand the clinical characteristics of our sample. We then ran separate multiple linear regression analyses for each MFIS score (total, physical, cognitive, and psychosocial) as a continuous measure of fatigue. For said regressions, neuropsychiatric predictors included scores from the HADS (anxiety and depression) and the FrSBe (apathy, disinhibition, executive dysfunction), with higher scores on both measures indicating higher levels of symptom severity. Cognitive predictors included in regressions were comprised of *T* scores from neuropsychological tests described above, with the exception of T-MoCA scores which were raw scores. Lower *T* score values indicate poorer performance on the test, with *T* scores lower than 36 considered as “below average” and those lower than 30 as “exceptionally low scores,” according to the ranges proposed by the American Academy of Clinical Neuropsychology [34]. CPT-II scores were interpreted in the opposite fashion, with higher *T* scores indicating poorer performance and scores higher than 60 considered to be clinically significant. In all analyses, we included the following predictors as being possible confounding variables: days of hospitalization, days elapsed between diagnosis and testing, and age (years).

We employed a stepwise regression method, consisting of adding and removing predictors according to a level of significance of 0.05. Variances explained by predictors are reported for each model (*R*^2^) and partial regression coefficients are reported when predictors were found to be significant. We checked for multicollinearity by inspecting the variance inflation factor (VIF), which measures the strength of correlation between predictors in a regression model. A cutoff value of 4 was used in determining the presence of collinearity [35].

## Results

### Fatigue

112 patients (82.3%) showed clinically significant levels of fatigue according to their MFIS scores (total score: *M* = 57.9, SD = 11.7; cognitive subscale score (max. 40): *M* = 26.5, SD = 6.4; physical subscale score (max. 36): *M* = 25.9, SD = 5.8; psychosocial subscale score (max. 8): *M* = 5.3, SD = 1.9). This fatigue group showed worse quality of life and poor daily functioning (EQ-5D: *M* = 8.9, SD = 2.3; BBQ: *M* = 50.2, SD = 20.3; WHOQOL-BREF-domain 1: *M* = 39.9, SD = 16.2; WHOQOL-BREF-domain 2: *M* = 45.1, SD = 18.0; WHOQOL-BREF-domain 3: *M* = 49.7, SD = 17.9) as compared to those who were classified without fatigue (EQ-5D: *M* = 6.7, SD = 0.9; BBQ: *M* = 62.8, SD = 15.6; WHOQOL-BREF-domain 1: *M* = 59.8, SD = 18.3; WHOQOL-BREF-domain 2: *M* = 62.8, SD = 19.9; WHOQOL-BREF-domain 3: *M* = 67.3; SD = 21.1, *p* < 0.001).

### Depression, anxiety, apathy, disinhibition, and executive functioning

Based on HADS scores, thirty-two patients (23.5%) showed clinically significant levels of depressive symptoms, while 48 (35.3%) patients showed significant levels of anxiety.

There were significant changes between retrospective “before COVID” scores and current state scores of the FrSBe. For the total score, the number of patients with scores meeting the cutoff for significant front systems dysfunction increased from 27 (19.9%) before COVID to 93 (68.4%) at the time of assessment (*χ*^2^(1) = 51.06, *p* < 0.001). For the apathy subscale, this same increase was from 23 (16.9%) to 85 patients (62.5%) (*χ*^2^(1) = 73.65, p < 0.001). For the disinhibition subscale, the increase was from 25 (18.4%) to 53 patients (38.9%) (*χ*^2^(1) = 14.09, *p* < 0.001). For the executive dysfunction scale, the increase was from 35 (25.7%) to 83 patients (61.1%) (*χ*^2^(1) = 34.48, *p* < 0.001). (See Supplementary Materials for results split by patients with and without fatigue).

### Neuropsychological deficits

Ninety-five (69.8%) patients scored below the cutoff (19) on the T-MoCA. The frequency of scores in the below average score and exceptionally low score ranges (PC < 8, or *T* < 36; [34]) are reported per cognitive domains described in García-Sánchez et al. [27]’s study (See Supplementary Materials for results split by patients with and without fatigue):

- Language (*n* = 136): 6 (4.4%) on the BNT, 25 (18.3%) on semantic fluency, and 23 (16.9%) on phonological fluency.
- Learning and Long-Term Memory (*n* = 136): 44 (32.3%) on RAVLT-Immediate recall, 39 (28.7%) on RAVLT-Delayed recall, and 23 (16.9%) on ROCFT-Delayed recall:
- Visuospatial and Visuoconstructive Abilities (n = 136): 10 (7.3%) on the Block Design Test and 14 (10.3%) on the ROCFT—Copy.
- Attention (*n* = 136): Scores from the CPT-II (see factorial analysis, Garcia-Sanchez et al. 2022). Omissions: 50 (36.8%); Commissions: 29 (21.3%); Variability: 56 (41.2%); Hit RT: 49 (36.1%); Detectability: 27 (19.8%); Perseveration: 56 (41.2%); Hit SE: 37 (27.2%); Hit RT ISI Change: 43 (31.6%).
- Executive Functioning (EF) (*n* = 132): 37 (28.0%) on Stroop task-Reading, 51 (38.6%) on Stroop task-Color, 32 (24.2%) on Stroop task-Inhibition, 22 (16.7%) on Trail Making Test A, and 31 (23.5%) on Trail Making Test B.
- Processing speed (*n* = 136): 10 (7.5%) on Coding Test and 8 (5.9%) on Symbol Search test.

### Regression analyses

**Fig. 1:**
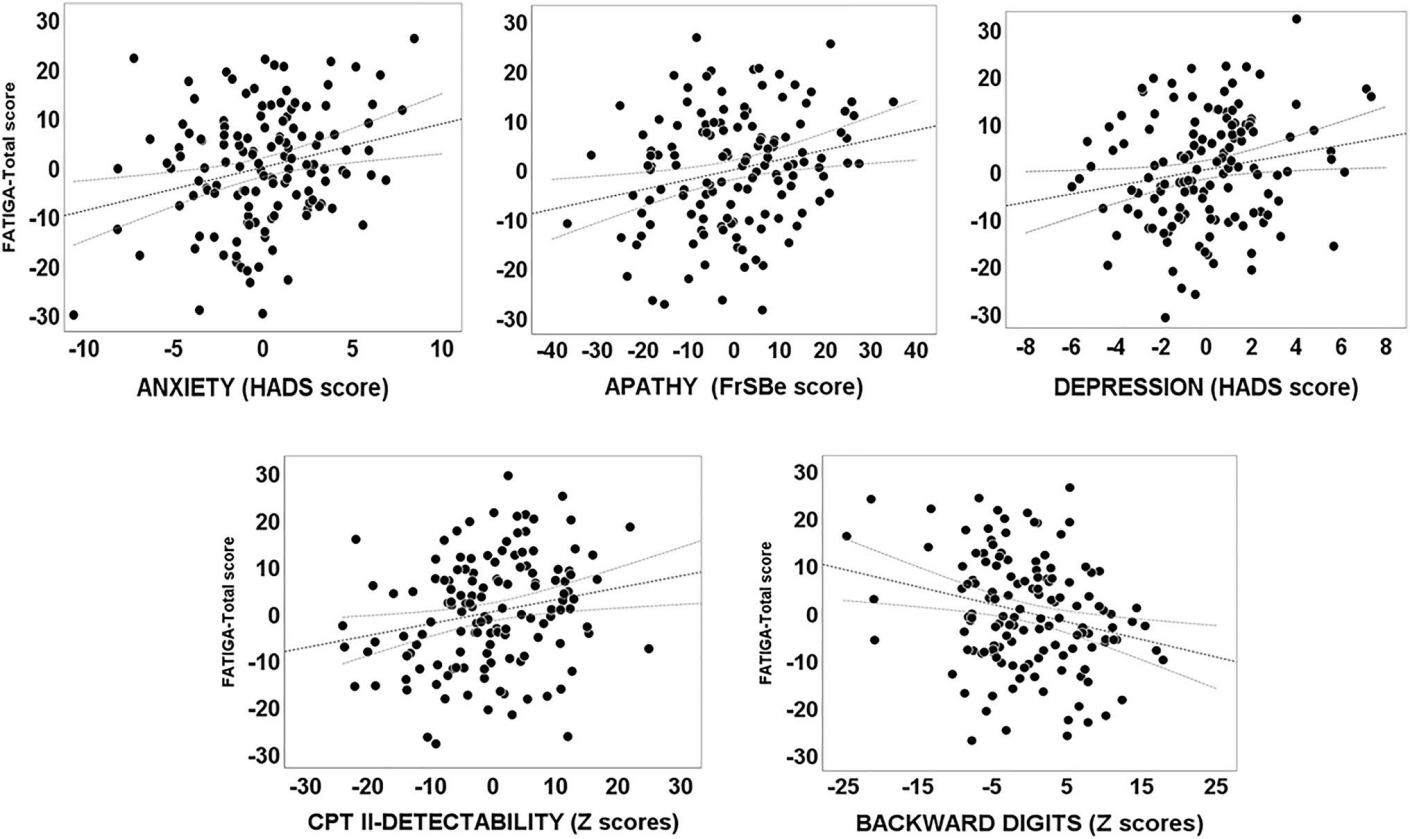
Partial regression plots for the significant effects of cognition and neuropsychiatric symptoms on total score of fatigue

For *the total score* on the MFIS, the best model, explaining 50.2% of the variance (*F*(5, 126) = 25.38, *p* < 0.001), included the following predictors: depression (HADS), anxiety (HADS), apathy, backward digits, and CPT-2 (Detectability) (Model 5, Table 1; for partial regression plots, see Fig. 1). VIFs did not suggest the presence of multicollinearity between these predictors: Depression = 2.69; Apathy (FrSBe) = 2.11; Anxiety = 1.89; Backward digits = 1.05; CPT-II (Detectability) = 1.05.

**Table 1:** Results of the regression analysis for the total score on the Modified Fatigue Impact Scale (MFIS)

Model	*R*	*R*^2^	Variables	*B*	SE	*β*	*t*	*p*	Partial correlations
**1**	0.603	0.363	(Intercept)	34.420	2.371		14.520	< 0.001	
			Depression (HADS)	2.452	0.284	0.603	8.628	< 0.001	0.603
**2**	0.648	0.419	(Intercept)	20.470	4.558		4.491	< 0.001	
			Depression (HADS)	1.472	0.389	0.362	3.784	< 0.001	0.254
			Apathy (FrSBe)	0.282	0.080	0.338	3.530	< 0.001	0.237
**3**	0.671	0.450	(Intercept)	19.505	4.475		4.358	< 0.001	
			Depression (HADS)	0.946	0.431	0.233	2.195	0.030	0.144
			Apathy (FrSBe)	0.248	0.079	0.298	3.141	0.002	0.206
			Anxiety (HADS)	0.823	0.316	0.234	2.600	0.010	0.171
**4**	0.692	0.479	(Intercept)	36.235	7.634		4.746	< 0.001	
			Depression (HADS)	0.819	0.424	0.202	1.933	0.055	0.124
			Apathy (FrSBe)	0.236	0.077	0.283	3.049	0.003	0.195
			Anxiety (HADS)	0.891	0.310	0.253	2.872	0.005	0.184
			Backward digits	− 0.320	0.120	– 0.174	– 2.673	0.009	– 0.170
**5**	0.703	0.502	(Intercept)	24.087	9.013		2.672	0.009	
			Depression (HADS)	0.800	0.416	0.197	1.924	0.057	0.121
			Apathy (FrSBe)	0.216	0.076	0.259	2.823	0.006	0.178
			Anxiety (HADS)	0.878	0.304	0.250	2.885	0.005	0.181
			Backward digits	− 0.297	0.118	– 0.162	– 2.524	0.013	– 0.159
			CPT-II (Detectability)	0.245	0.101	0.156	2.424	0.017	0.152

For the *cognitive subscale score* of the MFIS, the best model, explaining 41.8% of the variance (*F*(4, 127) = 22.88, *p* < 0.001), included the following predictors: depression (HADS), anxiety (HADS), executive dysfunction (FrSBe), and backward digits (Model 4, Table 2). VIFs did not suggest the presence of multicollinearity between these predictors: Depression = 2.04; Anxiety = 1.98; Executive Dysfunction (FrSBe) = 1.49; Backward digits = 1.03.

**Table 2:** Results of the regression analysis for the cognitive subscale score of the Modified Fatigue Impact Scale (MFIS)

Model	*R*	*R*^2^	Variables	*B*	SE	*β*	*t*	*p*	Partial correlations
**1**	0.553	0.306	(Intercept)	16.025	1.237		12.955	< 0.001	
			Depression (HADS)	1.119	0.148	0.553	7.541	< 0.001	0.553
**2**	0.611	0.373	(Intercept)	7.783	2.522		3.086	0.002	
			Depression (HADS)	0.796	0.166	0.393	4.782	< 0.001	0.389
			Executive dysfunction (FrSBe)	0.149	0.040	0.304	3.698	< 0.001	0.259
**3**	0.630	0.397	(Intercept)	7.924	2.483		3.191	0.002	
			Depression (HADS)	0.549	0.197	0.271	2.789	0.006	0.192
			Anxiety (HADS)	0.388	0.171	0.220	2.268	0.025	0.156
			Executive dysfunction (FrSBe)	0.124	0.041	0.254	3.021	0.003	0.208
**4**	0.646	0.418	(Intercept)	14.677	4.030		3.642	< 0.001	
			Depression (HADS)	0.482	0.197	0.238	2.453	0.016	0.167
			Anxiety (HADS)	0.412	0.169	0.233	2.433	0.016	0.165
			Executive dysfunction (FrSBe)	0.124	0.041	0.253	3.048	0.003	0.207
			Backward digits	− 0.133	0.063	− 0.146	-2.110	0.037	−0.143

For the *physical subscale score* of the MFIS, the best model, explaining 40.8% of the variance (*F*(4, 127) = 21.68, *p* < 0.001), included the following predictors: apathy (FrSBe), anxiety (HADS), TMT-B, and sex (Model 4, Table 3). VIFs did not suggest the presence of multicollinearity between these predictors: Apathy (FrSBe) = 1.52; Anxiety (HADS) = 1.50; TMT-B = 1.03; Sex = 1.06.

**Table 3:** Results of the regression analysis for the physical subscale score of the Modified Fatigue Impact Scale (MFIS)

Model	*R*	*R*^2^	Variables	*B*	SE	*β*	*t*	*p*	Part. Corr
**1**	0.544	0.296	(Intercept)	6.176	2.392		2.582	0.011	
			Apathy (FrSBe)	0.228	0.031	0.544	7.399	< .001	0.544
**2**	0.594	0.353	(Intercept)	4.854	2.339		2.075	0.040	
			Apathy (HADS)	0.210	0.030	0.500	6.925	< .001	0.491
			Sex	4.142	1.247	0.240	3.323	0.001	0.236
**3**	0.623	0.389	(Intercept)	13.482	3.856		3.496	< 0.001	
			Apathy (HADS)	0.198	0.030	0.471	6.629	< 0.001	0.458
			Sex	3.996	1.217	0.231	3.285	0.001	0.227
			TMT-B	– 0.180	.065	– .194	– 2.775	0.006	– 0.192
**4**	0.629	0.408	(Intercept)	14.087	3.821		3.686	< .001	
			Apathy (FrSBe)	0.159	0.035	0.379	4.527	< .001	0.309
			Anxiety (HADS)	0.301	0.148	0.170	2.032	0.044	0.139
			TMT-B	– 0.182	0.064	– 0.197	– 2.850	0.005	– 0.195
			Sex	3.604	1.217	0.209	2.961	0.004	0.202

For the *psychosocial subscale score* of the MFIS, the best model, explaining 42.7% of the variance (*F*(3, 128) = 32.56, *p* < 0.001), included the following predictors: apathy (HADS), depression (HADS), and executive dysfunction (FrSBe) (Model 3, Table 4). VIFs did not suggest the presence of multicollinearity between these predictors: Apathy (FrSBe) = 2.88; Depression = 2.05; Executive Dysfunction (FrSBe) = 1.95].

**Table 4:** Results of the regression analysis for the psychosocial subscale score of the Modified Fatigue Impact Scale (MFIS)

Model	*R*	*R*^2^	Variables	*B*	SE	*β*	*t*	*p*	Partial correlations
1	0.595	0.354	(Intercept)	– 0.118	0.608		– 0.194	0.846	
			Apathy (FrSBe)	0.066	0.008	0.595	8.335	< 0.001	0.595
2	0.625	0.391	(Intercept)	0.814	0.681		1.236	0.219	
			Apathy (FrSBe)	0.087	0.011	0.782	8.078	< 0.001	0.561
			Executive dysfunction (FrSBe)	– 0.035	0.013	– 0.270	– 2.784	0.006	– 192
3	0.653	0.427	(Intercept)	1.414	0.696		2.031	0.044	
			Apathy (FrSBe)	0.066	0.013	0.598	5.184	< 0.001	0.351
			Depression (HADS)	0.145	0.052	0.270	2.779	0.006	0.190
			Executive dysfunction (FrSBe)	– 0.037	0.012	– 0.272	– 2.927	0.005	– 0.202

## Discussion

This study was conceived to further characterize the relationship between fatigue and cognitive/neuropsychiatric symptoms in post-COVID-19 patients. To investigate this relationship, the present study explored how symptoms of depression, anxiety, and apathy, as well as neuropsychological scores across several cognitive domains, may contribute to different areas of fatigue (cognitive, physical, and psychosocial). We uncovered several findings indicating that both cognitive and neuropsychiatric symptoms contribute to post-COVID-19 fatigue in complex ways.

First, descriptive analysis provided general insight into our sample and their cognitive functioning, emotional states, and everyday functioning. Fatigue was very prevalent in our sample, as 82.3% of individuals reported clinically significant levels of fatigue on the MFIS. This exceeds the prevalence reported in some reviews that estimated fatigue in about 30% of COVID-19 patients [9–11]. This discrepancy might be explained by the characteristics of our sample, which included COVID-19 patients with subjective cognitive complaints. While these complaints were not further specified, it is possible that many of these individuals’ complaints consisted of persistent fatigue months after infection. Furthermore, we found that patients with clinically significant fatigue showed worse quality of life and poor daily functioning, reflected in scores on the EQ-5D, the BBQ, and the WHOQOL-BREF. Depressive symptoms and anxiety were less prevalent in our sample, with 23.5% and 35.3% of patients reaching significant levels, respectively. Interestingly, apathy and executive dysfunction measured by the FrSBe consistently increased after infection. Apathy increased the most among the three FrSBe subscales, with 16.9% of patients reaching cutoff before COVID and 62.5% of individuals at the time of assessment. Patients reporting significant levels of executive dysfunction increased from 25.7 to 61.1% of the sample. The most prevalent neuropsychological deficits were in long-term memory (28.7%), executive functioning (Stroop Inhibition: 24.2%; TMT-B: 23.5%), and attention as measured by CTP-II (especially for perseverations and variability). These results are consistent with our previous findings [27] in a sample of COVID-19 patients with subjective cognitive complaints, as well as findings from other studies that investigated neuropsychological status after infection (see reviews, [36, 37].

Second, we found that the three components of fatigue were predicted by different factors. Measured by the MFIS total score, overall fatigue was largely predicted by depression, apathy, anxiety and performance on two cognitive tests of executive control (working memory) and sustained attention. That is, the more severe affective symptomatology and attention/executive impairments were, the higher the total fatigue score was. These predictors explained half of the total variance, with each component displaying a similar contribution (see partial correlations). In contrast, the cognitive component of fatigue was most strongly predicted by neuropsychiatric symptoms (levels of depression and anxiety), executive dysfunction, and cognitive deficits in working memory. Compared to both physical and psychosocial components of fatigue, apathy was the strongest predictor of fatigue among significant measures in models. For the physical component, executive control deficits (switching abilities) and anxiety were also shown to be strong, significant predictors. Interestingly, sex was also a significant predictor for this component, suggesting that being female is associated with higher scores of physical fatigue compared to being males. We acknowledge that this effect might be related to the atypical distribution of men and women in our sample. Indeed, there were more females (64%) than males (36%) in our sample, whereas epidemiological studies report that COVID-19 is more frequent in men (55%) than in women (45%) [38].

Finally, the psychosocial component was also predicted by depressive symptomatology and executive dysfunction. Of note, the relationship between executive dysfunction and fatigue was somewhat counterintuitive, with lower executive dysfunction scores (i.e., higher reported executive functioning) predicting higher scores of fatigue. A possible explanation for this finding is that the two items that assessed this psychosocial fatigue refer to social contact and performing activities outside of home, both activities that were limited by self-isolation during the pandemic. Therefore, it may be that the scores on this subscale were distorted by the context-driven restriction and not by fatigue, per se.

In summary, it appears that both cognitive and neuropsychiatric symptoms contribute to the different dimensions of fatigue in this population. Our results are in line with the findings about the role of apathy [22, 23], depression and anxiety [10, 12] on fatigue. Executive functioning and attention were shown here to be crucial cognitive factors related to fatigue, similar to findings from previous studies [16, 17, 26]. These results highlight the importance of assessing both neuropsychiatric and cognitive symptoms as clinical variables that partially explain post-COVID-19 fatigue.

Finally, it is important to comment on the influence of socio-demographic variables and disease severity on fatigue. Sex was found to be a significant predictor in only the physical component of fatigue, so we might conclude that sex is not a strong factor for determining generalized fatigue. Interestingly, several other clinical variables associated with the severity of infection were not significant predictors of the magnitude of fatigue suffered by COVID-19 patients, replicating, as found in a previous study by Townsend et al. [20]. These included time elapsed between infection and assessment, as well as the number of days hospitalized. This means that early post-infection fatigue persists with the same characteristics over time and across levels of disease severity in the acute phase of COVID-19 infection. Hence, specific treatment interventions for this long-term condition of COVID-19 are needed to reduce the impact on daily functioning and quality of life. The lack of a significant effect of hospitalization was also found in the reviews on fatigue by Mazza et al. [11] and Ceban et al. [9]. These authors reported that the number of patients who reported fatigue after infection was the same, regardless of whether they were hospitalized or not. Similarly, in our previous study [27], we found that hospitalization did not affect the prevalence and severity of cognitive symptoms after infection. Thus, these results suggest that mild, as well as more severe, symptomatology associated with SARS-CoV-2 infection may have negative consequences, such as persistent fatigue and cognitive impairment.

We acknowledge that our study has some limitations. First, we did not compare our sample with a healthy group of individuals without COVID-19 or a group of COVID-19 patients without subjective cognitive complaints. However, the use of normative data allowed us to draw conclusions about the presence of neuropsychiatric symptoms and cognitive deficits, thus lending support to the validity of our findings. Second, we acknowledge that our findings cannot be generalized to all patients who were diagnosed with COVID-19, only to those who report subjective cognitive complaints. Nevertheless, we believe that our results are clinically relevant for the understanding of post-COVID-19 fatigue and for neuropsychologists assessing cognition in COVID-19 patients who report lasting effects of the disease.

## Supplementary Information

Below is the link to the electronic supplementary material.

Supplementary file1 (DOCX 30 KB)
