# Supplementary material for: Post-COVID-19 fatigue: the contribution of cognitive and neuropsychiatric symptoms
Source: J Neurol. 2022 Apr 30;269(8):3990–9. doi: 10.1007/s00415-022-11141-8 (PMC9055007; doi:10.1007/s00415-022-11141-8)
Supplement: Supplementary file 1 — Supplementary file1 (DOCX 30 KB) [file 415_2022_11141_MOESM1_ESM.docx]

**Article title:** Post COVID-19 fatigue: the contribution of cognitive and neuropsychiatric symptoms

**Supplemental Material – Neuropsychological Tests and Normative Data**

1. *Telephone MoCA (T-MoCA)*[1]*,* a telephone version of the *Montreal Cognitive Assessment* [2] (MoCA)*,* a screening tool for cognitive impairment.
2. *Conners Continuous Performance Test II* [3] *(CPT-II)*, which measures sustained attention.
3. *Rey's Auditory Verbal Learning Test* [4] *(RAVLT)*, a test of verbal long-term memory and learning;
4. *Rey-Osterrieth Complex Figure Test* [5–7] (ROCFT), which measures visuoconstructive abilities (copy) and non-verbal long-term memory (delayed recall);
5. *Digit Span Backward and Digit Span Forward*  [8–10]*,* which measure short-term and working memory;
6. *Boston Naming Test* [11–13], that assesses picture naming abilities;
7. *Block Design Test,* a subtest from WAIS-IV [8], which measures visuoconstructive functions;
8. *Coding Test*, one of two subtests in the Processing Speed Index from WAIS-IV [8], which measures processing speed by way of visual-motor coordination;
9. *Symbol Search,* the other subtest in the Processing Speed Index from WAIS-IV [8], which measures processing speed in addition to visual discrimination;
10. *Trail Making Tests* [9, 10, 14], which measure the visual attention (part A) and task switching (part B);
11. *Stroop task* [15–17]*,* which measures inhibitory control and verbal interference;
12. *Phonetic and semantic fluency tasks* [18, 19]*,* which measure verbal fluency;

**Sociodemographic, clinical, neuropsychological, neuropsychiatric, and quality of life**

**data for patients with and without fatigue**

Scores of the neuropsychological tests were scored as “below average” and clinically significant if the T scores was lower than 36, following the ranges proposed by the American Academy of Clinical Neuropsychology [20]. Likewise, CPT-II scores higher than 60 were considered to be clinically significant.

The scores of the neuropsychiatric tests, quality of life and impact on daily functioning questionnaires were considered to be clinically significant according to test-specific cutoffs.

| **Variables** | **Patients with fatigue**  **(n=112)** | **Patients without fatigue**  **(n= 24)** |
| --- | --- | --- |
| **Sociodemographic and clinical data** | **Mean (SD)** | |
| Age | 51.8 (13.5) | 51.2 (15.1) |
| Education (years) | 13.6 (3.2) | 15.0 (3.4) |
| Disease duration (days) | 249.4 (146.5) | 256.1 (165.2) |
| Hospitalization (days) | 8.0 (14.8) | 10.1 (17.1) |
| Sex (Males/Females) | 36/76 | 13/11 |
| **Neuropsychological assessment** | **Number of clinically significant cases (%)** | |
| **T-MoCA** | 80 (71.4) | 15 (62.5) |
| **Long-term memory** |  |  |
| RAVLT - Trial I | 35 (31.3) | 10 (41.7) |
| RAVLT - Trial V | 28 (25.0) | 8 (33.3) |
| RAVLT -Total | 35 (31.3) | 9 (37.5) |
| RAVLT - Delayed recall | 29 (25.9) | 9 (37.5) |
| RAVLT - Recognition | 24 (21.4) | 7 (29.2) |
| ROCFT - Delayed recall | 22 (19.6) | 1 (4.2) |
| **Visuospatial and visuoconstructional abilities** |  |  |
| ROCFT - Copy | 13 (11.6) | 1 (4.2) |
| ROCFT - Time | 13 (11.6) | 0 (0.0) |
| WAIS-IV - Block Design | 10 (8.9) | 0 (0.0) |
| **Short-term and working memory** |  |  |
| Forward Span | 27 (24.1) | 4 (16.7) |
| Backward Span | 6 (5.4) | 0 (0.0) |
| Processing speed |  |  |
| WAIS-IV - Coding Test | 9 (8.0) | 1 (4.2) |
| WAIS-IV - Symbol Search | 7 (6.3) | 1 (4.2) |
| **Language** |  |  |
| Boston Naming | 6 (5.4) | 0 (0.0) |
| Phonemic fluency | 18 (16.1) | 5 (20.8) |
| Semantic fluency | 22 (19.6) | 3 (12.5) |
| **Sustained attention** |  |  |
| CPT-II - Omissions % | 44 (39.3) | 6 (25.0) |
| CPT-II - Commissions % | 26 (23.2) | 3 (12.5) |
| CPT-II - Hit RT | 39 (34.8) | 10 (41.7) |
| CPT-II - Hit SE | 67 (59.8) | 11 (45.8) |
| CPT-II - Variability | 49 (43.8) | 7 (29.2) |
| CPT-II - Detectability (d') | 24 (21.4) | 3 (12.5) |
| CPT-II - Response Style (β) | 19 (17.0) | 3 (12.5) |
| CPT-II - Perseverations % | 50 (44.6) | 6 (25.0) |
| CPT-II - Hit RT Block Change | 21 (18.8) | 5 (20.8) |
| CPT-II - Hit SE Block Change | 31 (27.7) | 4 (16.7) |
| CPT-II - Hit RT ISI Change | 34 (30.4) | 9 (37.5) |
| CPT-II- Hit SE ISI Change | 32 (28.6) | 5 (20.8) |
| **Executive functioning** |  |  |
| TMT-A | 21 (18.8) | 1 (4.2) |
| TMT-B | 27 (24.1) | 2 (8.3) |
| Stroop-Reading | 33 (29.5) | 4 (16.7) |
| Stroop-Color | 45 (40.2) | 5 (20.8) |
| Stroop-Inhibition | 28 (25.0) | 4 (16.7) |
| **Neuropsychiatric symptoms** | **Number of clinically significant cases (%)** | |
| Anxiety (HADS) | 47 (42.0) | 1 (4.2) |
| Depression (HADS) | 31 (27.7) | 1 (4.2) |
| Apathy-before (FrSBe) | 22 (19.6) | 1 (4.2) |
| Apathy-present (FrSBe) | 86 (76.8) | 7 (29.2) |
| Disinhibition-before (FrSBe) | 23 (20.5) | 2 (8.4) |
| Disinhibition-present (FrSBe) | 48 (42.9) | 5 (20.8) |
| Executive dysfunction-before (FrSBe) | 31 (27.7) | 4 (16.7) |
| Executive dysfunction-present (FrSBe) | 75 (67.0) | 8 (33.3) |
| Total score FrSBe-before | 26 (23.2) | 1 (4.2) |
| Total score FrSBe-present | 78 (69.6) | 7 (29.2) |
| **Measures of quality of life and impact on daily functioning** | **Number of clinically significant cases (%)** | |
| European Quality of Life-5 Dimensions (EQ-5D) | 101 (90.2) | 19 (79.2) |
| Brunnsviken Brief Quality of life scale (BBQ) | 62 (50.4) | 5 (20.8) |
| World Health Organization Quality of Life – BREF- domain 1 | 98 (87.4) | 10 (42.1) |
| World Health Organization Quality of Life – BREF- domain 2 | 92 (82.1) | 11 (47.4) |
| World Health Organization Quality of Life – BREF- domain 3 | 86 (76.8) | 7 (31.6) |
| World Health Organization Quality of Life – BREF- domain 4 | 51 (45.3) | 6 (26.3) |

**References**

1. Katz MJ, Wang C, Nester CO, et al (2021) T-MoCA: A valid phone screen for cognitive impairment in diverse community samples. Alzheimer’s Dement Diagnosis, Assess Dis Monit 13:. https://doi.org/10.1002/dad2.12144

2. Nasreddine ZS, Phillips NA, Bédirian V, et al (2005) The Montreal Cognitive Assessment, MoCA: A brief screening tool for mild cognitive impairment. J Am Geriatr Soc 53:695–699. https://doi.org/10.1111/j.1532-5415.2005.53221.x

3. Conners CK (2000) Conners’ Continuous Performance Test II: Technical guide

4. Savage RM, Gouvier WD (1992) Rey auditory-verbal learning test: The effects of age and gender, and norms for delayed recall and story recognition trials. Arch Clin Neuropsychol 7:407–414. https://doi.org/10.1016/0887-6177(92)90153-E

5. Osterrieth PA (1944) Le test de copie d’une figure complexe; contribution à l’étude de la perception et de la mémoire. Arch Psychol (Genève) 30:206–356

6. Palomo R, Casals-Coll M, Sánchez-Benavides G, et al (2013) Estudios normativos españoles en población adulta joven (proyecto NEURONORMA jóvenes): Normas para las pruebas Rey-Osterrieth Complex Figure (copia y memoria) y Free and Cued Selective Reminding Test. Neurologia 28:226–235. https://doi.org/10.1016/j.nrl.2012.03.008

7. Peña-Casanova J, Gramunt-Fombuena N, Quiñones-Úbeda S, et al (2009) Spanish multicenter normative studies (NEURONORMA project): Norms for the rey-osterrieth complex figure (copy and memory), and free and cued selective reminding test. Arch Clin Neuropsychol 24:371–393. https://doi.org/10.1093/arclin/acp041

8. Campos A (2013) Escala de inteligencia de Wechsler para adultos-IV (WAIS-IV). Univ Barcelona 1–21

9. Tamayo F, Casals-Coll M, Sánchez-Benavides G, et al (2012) Spanish normative studies in a young adult population (NEURONORMA young adults project): Guidelines for the span verbal, span visuo-spatial, Letter-Number Sequencing, Trail Making Test and Symbol Digit Modalities Test. Neurol (English Ed 27:319–329. https://doi.org/10.1016/j.nrleng.2012.07.008

10. Peña-Casanova J, Quiñones-Úbeda S, Quintana-Aparicio M, et al (2009) Spanish Multicenter Normative Studies (NEURONORMA Project): Norms for Verbal Span, Visuospatial Span, Letter and Number Sequencing, Trail Making Test, and Symbol Digit Modalities Test. Arch Clin Neuropsychol 24:321–341. https://doi.org/10.1093/arclin/acp038

11. Kaplan EF, Goodglass H, Weintraub S (2001) The Boston Naming Test (ed. 2). Philadelphia Lea Febiger

12. Aranciva F, Casals-Coll M, Sánchez-Benavides G, et al (2012) Spanish normative studies in a young adult population (NEURONORMA young adults project): Norms for the Boston Naming Test and the Token Test. Neurol (English Ed 27:394–399. https://doi.org/10.1016/j.nrleng.2011.12.010

13. Peña-Casanova J, Quiñones-Úbeda S, Gramunt-Fombuena N, et al (2009) Spanish multicenter normative studies (NEURONORMA project): Norms for boston naming test and token test. Arch Clin Neuropsychol 24:343–354. https://doi.org/10.1093/arclin/acp039

14. Bowie CR, Harvey PD (2006) Administration and interpretation of the Trail Making Test. Nat Protoc 1:2277–2281. https://doi.org/10.1038/nprot.2006.390

15. Stroop JR (1935) Studies of interference in serial verbal reactions. J Exp Psychol 18:643–662. https://doi.org/10.1037/h0054651

16. Peña-Casanova J, Quiñones-Úbeda S, Gramunt-Fombuena N, et al (2009) Spanish multicenter normative studies (NEURONORMA project): Norms for the stroop color-word interference test and the tower of London-Drexel. Arch Clin Neuropsychol 24:413–429. https://doi.org/10.1093/arclin/acp043

17. Rognoni T, Casals-Coll M, Sánchez-Benavides G, et al (2013) Spanish normative studies in young adults (NEURONORMA young adults project): Norms for Stroop Color–Word Interference and Tower of London-Drexel University tests. Neurol (English Ed 28:73–80. https://doi.org/10.1016/j.nrleng.2012.02.004

18. Casals-Coll M, Sánchez-Benavides G, Quintana M, et al (2013) Spanish normative studies in young adults (NEURONORMA young adults project): Norms for verbal fluency tests. Neurol (English Ed 28:33–40. https://doi.org/10.1016/j.nrleng.2012.02.003

19. Peña-Casanova J, Quiñones-Úbeda S, Gramunt-Fombuena N, et al (2009) Spanish multicenter normative studies (NEURONORMA project): Norms for verbal fluency tests. Arch Clin Neuropsychol 24:395–411. https://doi.org/10.1093/arclin/acp042

20. ​​Guilmette TJ, Sweet JJ, Hebben N, et al (2020) American Academy of Clinical Neuropsychology consensus conference statement on uniform labeling of performance test scores. Clin Neuropsychol 34:437–453. https://doi.org/10.1080/13854046.2020.1722244
